# Diffusion Kurtosis Imaging of Microstructural Changes in Gray Matter Nucleus in Parkinson Disease

**DOI:** 10.3389/fneur.2020.00252

**Published:** 2020-04-17

**Authors:** Gao Bingbing, Zhou Yujing, Miao Yanwei, Dong Chunbo, Wang Weiwei, Tian Shiyun, Liu Yangyingqiu, Shang Jin, Song Qingwei, Liu Ailian, Xie Lizhi

**Affiliations:** ^1^Department of Radiology, First Affiliated Hospital of Dalian Medical University, Dalian, China; ^2^GE Healthcare, MR Research, Beijing, China

**Keywords:** parkinson disease, diffusion magnetic resonance imaging, gray matter, extrapyramidal tracts, neurodegenerative diseases

## Abstract

**Objective:** To explore the microstructural damage of extrapyramidal system gray matter nuclei in Parkinson disease (PD) using diffusion kurtosis imaging (DKI).

**Materials and Methods:** We enrolled 35 clinically confirmed PD patients and 23 healthy volunteers. All patients underwent MR examination with conventional MRI scan sequences and an additional DKI sequence. We subsequently reconstructed the DKI raw images and analyzed the data. A radiologist in our hospital collected the Mini-Mental State Examination (MMSE) score of all subjects.

**Results:** In the PD group, the mean kurtosis and axial kurtosis level decreased in the red nucleus (RN) and thalamus; the radial kurtosis increased in the substantia nigra (SN) and globus pallidus (GP). Fractional anisotropy decreased in the putamen. The largest area under the ROC curve of mean diffusion in GP was 0.811. Most kurtosis parameters demonstrated a positive correlation with the MMSE score, while several diffusion parameters showed a negative correlation with the same.

**Conclusion:** DKI can qualitatively distinguish PD from healthy controls; furthermore, DKI-derived parameters can quantitatively evaluate the modifications of microstructures in extrapyramidal system gray matter nucleus in PD.

## Introduction

Parkinson disease (PD) is the second most common neurodegenerative disease. Clinical diagnosis of PD is based on motor symptoms and signs including rest tremor, bradykinesia, rigidity and postural instability, and several variations of clinical scales. However, these symptoms are not particularly exclusive to PD, considering the fact that a number of other diseases have similar symptoms or atypical parkinsonian disorders. Due to this, the early diagnosis of PD is particularly difficult in clinical settings. This issue can be addressed by radiologists, by exploring the structural and functional changes in the brain of PD patients via various MR protocols. A previous study reported that neurons projecting from lateral nigra to posterior dorsal putamen were most severely affected by Lewy body pathology in PD (1). Volumetric analysis of basal ganglia nuclei and SN in patients with PD revealed tissue atrophy at the cortical, basal ganglia, and brainstem (particularly the SN and red nucleus, RN) levels (2); furthermore, the motor nerve conduction among these were divided into the pyramidal and extrapyramidal systems. Pathophysiology and pathology of PD demonstrated that the diminution of the brainstem is attributed to the neurotic breakdown and necrosis in SN, which indicated the existence of microstructural changes inside the nucleus. Pathological studies also reported the presence of neurotic changes at levels of the cortex and basal ganglia (2). We hypothesized that there must be a connection inside the nuclei of the extrapyramidal system. Diffusion kurtosis imaging (DKI) offers improved sensitivity to tissue microstructure (3), which may be considered as an advantage over diffusion tensor imaging (DTI) (4). The basal ganglia and brainstem can be visualized in the DKI images (5). We aimed to investigate microstructural changes of extrapyramidal system nuclei in PD patients and its association with the disease progression, and to explore the connection of nuclei exchange between the levels of the basal ganglia and brainstem.

## Materials and Methods

### Subjects

The prospective study was approved by local ethics committee, and each participant provided written consent. We enrolled 35 clinical diagnosed PD patients hospitalized in our hospital, along with 23 sex- and age-matched healthy controls (HC). PD was diagnosed based on the criteria provided by UK Parkinson Disease Society Brain Bank. The criteria also included the absolute exclusion criteria of PD, resulting in the exclusion of patients with hypertension and diabetes. The exclusion criteria for those in the HC group were history of surgery, cardiovascular disease, hypertension, diabetes, and other chronic diseases. The demographic and clinical data of the two groups are presented in Table 1. All patients and HC were right-handed. We assessed the Mini-Mental State Examination (MMSE) of all subjects; the MMSE score of all HC was above 27. Moreover, subjects with none or mild T2WI or T2 FLAIR high signal (Fazekas, grade 1) were considered normal. All subjects had no trauma, tumor, or a history of surgeries involving the central nervous system.

**Table 1:** Clinical data of PD and HC groups.

	**PD**	**HC**
*n*	35	23
Age (years), mean ± SD	67.00 ± 8.76	66.48 ± 5.20
Sex (M/F)	18/17	12/11
MMSE (score), mean ± SD	22.91 ± 4.27	29.91 ± 0.42
Course (years)	3–10	—

*All patients had been treated with dopamine agonists*.

### Images

Each subject underwent a GE Revolution CT scan of whole brain to exclude physiological or pathological calcification. All subjects underwent MR examination on a GE Signa HDxT 3.0 T MR scanner with a dedicated eight-channel head coil. The protocols included routine axial T1-, T2-, and diffusion kurtosis weighted images. DKI was obtained using an echo-planar imaging technique with *b* values of 0, 1,000, and 2,000 s/mm^2^ in 15 directions. Parameters of each protocol are described in Table 2. Scan baseline was parallel to the line running through the anterior commissure and the posterior commissure. Scan area ranged from the foramen magnum to the cranial vault.

**Table 2:** MRI protocol parameters.

**Protocol**	**TR**	**TE**	**TI**	**Thick slices**	**Intersection**	**FOV**	**Matrix size**
	**(ms)**	**(ms)**	**(ms)**	**(mm)**	**(mm)**	**(cm × cm)**	
T1WI	2,500	25	/	6	1	22.0 ×19.8	320 ×256
T2WI	5,000	118	/	6	1	22.0 ×19.8	320 ×256
T2 Flair	9,000	172	2250	6	1	22.0 ×22.0	256 ×192
DKI	10,000	107	/	4	0	24.0 ×24.0	128 ×128

### DKI Interpretation

#### Quantitative Assessment

Initial DKI images of all subjects were reconstructed on ADW4.4 workstation with Functool 2 software using all *b* values. The reconstructive images included mean kurtosis image (MK), axial kurtosis image (Ka), radial kurtosis image (Kr), mean diffusivity image (MD), axial diffusivity image (Da), radial diffusivity image (Dr), and fractional anisotropy image (FA), and the parameters were recorded as MK, Ka, Kr, MD, Da, Dr, and FA, respectively (Supplementary Table 2). Small round region of interest (ROI) was drawn with a diameter of 3 mm at the center of the maximum level of target nucleus (Supplementary Figure 1), which included RN, SN, thalamus (T), putamen (P), globus pallidus (GP), and the head of caudate nuclei (HCN). The ROIs were manually drawn by two neuroradiologists in the same manner. To improve intra-observer reliability, the neuroradiologists performed the same measurements for all subjects three times and average the results. To evaluate interobserver reliability, the intraclass correlation coefficients (ICCs) were calculated.

### Statistical Analysis

Demographic variables and MMSE scores were compared using two-tailed *t* tests and chi-squared tests. ICC test was calculated to evaluate interobserver reliability prior to data analysis. The consequent analysis would be performed if ICC test returned a consistent result (i.e., ICC ≥ 0.75). The diffusion and kurtosis parameters of right and left deep gray matter structures were compared using two-sided Satterthwaite *T* test. Two independent-samples *T* tests or two-sample Mann–Whitney *U* tests were used to compare the parameters of six nuclei between two groups. We performed the receiver operating characteristic (ROC) test to assess the ability of distinguishing two groups for each DKI parameter of every nucleus. The correlations between DKI parameters and MMSE score were tested using Spearman's correlation test. All measurement data were presented as mean value and standard deviation. *P* <0.05 was set to be statistically significant.

## Results

### Clinical Data

There were no observed significant differences between patient's age (*t* = 0.284, *P* = 0.777) and sex (χ^2^ = 0.069, *P* = 0.793) between the two groups. However, there was a significant intergroup difference in the MMSE score (*t* = 7.807, *P* = 0.000).

### DKI Data

**Figure 1:**
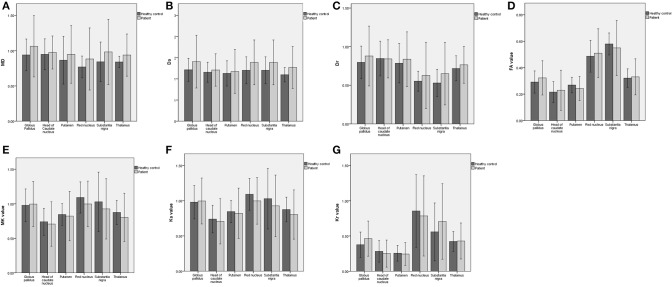
Comparison of DKI parameters between PD and HC groups.

The ICC values of all ROIs were >0.75 (shown in Supplementary Table 1). The results did not show any lateral difference in any of MK, Ka, Kr, MD, Da, Dr, and FA of each nucleus for all subjects (*P* > 0.05). Comparison of all the derived parameters between groups is presented in Figure 1.

### ROC Analysis

**Figure 2:**
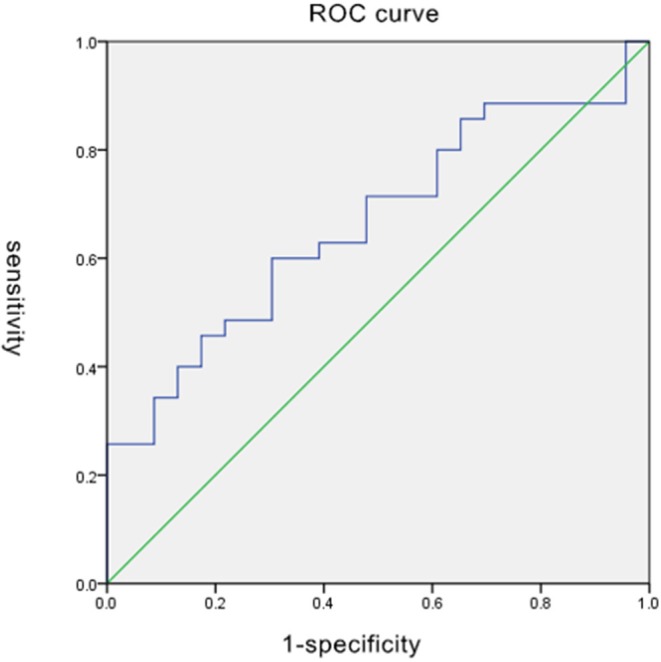
Sensitivity and specificity of MD of GP.

The largest area under ROC curve (AUC) value of 0.811 was observed for MD in GP. MD of GP had a sensitivity, specificity, and accuracy of 65.7% (23 out of 35), 87% (30 out of 35), and 81.1% (28 out of 35) when using a cutoff of 0.979 (shown in Table 3 and Figure 2).

**Table 3:** ROI analysis of DKI parameters of nuclei.

**Nucleus**	**Parameter**	**Accuracy**	**Cutoff**	**Sensitivity**	**Specificity**
**GP**	**MD**	**0.811**	**0.979**	**0.657**	**0.870**
RN	Da	0.796	1.285	0.686	0.783
SN	MD	0.775	0.900	0.714	0.826
GP	Da	0.763	1.450	0.457	**1.000**
SN	Da	0.742	1.560	0.714	0.826
SN	Dr	0.724	0.576	0.600	0.913
Putamen	MD	0.701	0.890	0.571	0.783

*The bold values are statistically significant results*.

### MMSE Score

**Figure 3:**
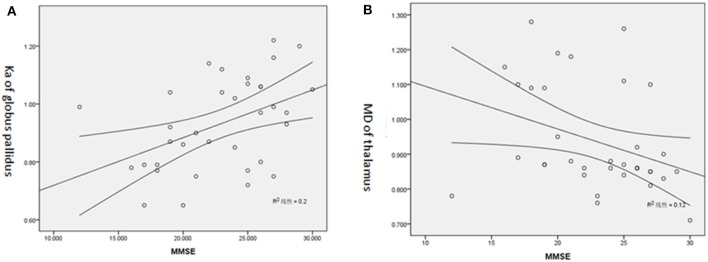
**(A)** Positive correlation of Ka of GP and MMSE score. **(B)** Negative correlation of MD of T and MMSE score.

The majority of the diffusion kurtosis parameters (MK, Ka, and Kr) demonstrated positive correlations with MMSE scores while the diffusion parameters exhibited several negative correlations (shown in Tables 4, 5 and Figure 3).

**Table 4:** The positive correlation of kurtosis parameters and MMSE scores.

**Score**	**Nucleus**		**MK value**	**Ka value**	**Kr value**
MMSE	Substantia nigra	*r*	0.284	**0.389**	0.130
		*P*	0.098	**0.021**	0.456
	Globus pallidus	*r*	**0.428**	**0.458**	**0.340**
		*P*	**0.010**	**0.006**	**0.046**
	Red nucleus	*r*	**0.353**	**0.337**	0.210
		*P*	**0.038**	**0.048**	0.226

*r refers to the correlation coefficient, P is 95% confidence (two-tailed)*.

*The bold values are statistically significant results*.

**Table 5:** The negative correlation of diffusion parameters and MMSE scores.

**Scores**	**Nucleus**		**MD value**	**Da value**	**Dr value**
MMSE	Thalamus	*r*	**−0.408**	**−0.384**	**–**0.241
		*P*	**0.015**	**0.023**	0.163
	Head of caudate nucleus	*r*	**−0.369**	**–**0.280	**−0.340**
		*P*	**0.029**	0.104	**0.045**
	Red nucleus	*r*	**–**0.091	**−0.354**	**–**0.043
		*P*	0.602	**0.037**	0.805

*r refers to the correlation coefficient, P is 95% confidence (two-tailed)*.

*The bold values are statistically significant results*.

## Discussion

DKI parameters provide a quantitative analysis of PD, and certain derived parameters offer novel diagnostic perspectives. A previous report revealed that DKI was highly sensitive to microstructural changes in tissue, which is not typically highlighted by the diffusion coefficient value (6). We observed statistical differences of DKI parameters between PD patients and HC, among which MD of GP was the best biomarker for diagnosis. It is well-recognized that DKI is sensitive to capture the “complexity” of tissue microstructure; thus, it has already been extensively used to probe microstructural changes in normal aging. The FA and MK of frontal lobe, temporal lobe, corpus callosum (knee and splenium), thalamus, posterior limb of internal capsule, and head of the caudate nucleus decreased with aging; however, there was an increase in the MD levels (7, 8). Another study by Latt, which focused on healthy adults across different age groups, observed that MK of thalamus, white matter of frontal lobe, knee of corpus callosum, and centrum semiovale were negatively linearly correlated with aging (9). Overall, a pattern of increased MD and radial and axial diffusivity, together with decreased FA, MK, and radial and axial kurtosis in GM, was found in normal aging (9–12). These age-related alterations may possibly be driven by mixed effects of axonal disintegration, cell loss, and iron accumulation. In our research, MD, Da, and Dr in some deep GM of PD group appeared to be higher than that of the HC group. However, a specific pattern of increased Kr in SN and GP was found in PD, which has the highest iron concentration in the brain in PD (13). Therefore, one probable interpretation of our result is excessive deposition of iron, neuronal degeneration, and apoptosis in SN and GP in PD patients, especially along the “radial” direction. We speculate that the alterations in diffusion metrics in PD may reflect both iron-related and age-related microstructure changes, with a leading factor by the former. This idea is supported by earlier research about effect of cocaine addiction and age on the microstructure of striatum and thalamus using DKI (14). Pathological studies of PD patients suggested that microstructural changes occur from brainstem at an early stage and subsequently spread to the limbic system and the cortices (15, 16). Therefore, the goal of this present study was to assess the microstructural changes in extrapyramidal system and to elucidate its associations.

### Diffusion and Kurtosis Parameters

Here, three diffusion parameters of the PD group appeared to be higher than that of the HC group. Studies have reported the presence of heterogeneous pathological encephalatrophy of brain in PD patients; additionally, the earliest onset was demonstrated in the SN and was considered as the most vulnerable structure (16). It was proposed that the dilated intercellular space caused by neuronal degeneration and apoptosis could enhance water molecule movement. Furthermore, the remaining areas of gray matter in PD patients may also experience a similar degree of modification. Damage in the neurons of the brainstem will lead to an accumulation of the materials in synapses of the nerve fibers transporting nutrients from cerebrum downward to the brainstem, resulting in the deficiency of nutritious materials in fibers from the brainstem downward to the spine. Both consequences will lead to secondary damage to adjacent structures and increase membrane permeability to facilitate the diffusion of water molecules.

Diffusion kurtosis parameters were associated with the complexity of structures, which varied across the diverse brain structures in healthy adults. The observed decline in the diffusion kurtosis parameters and FA may be due to neuronal degeneration and apoptosis combined with secondary damage of adjacent structures. Microglial cells are the most significant immune cells of brain. They distribute heterogeneously in brain, with their highest intensity being in the SN. In the resting (physiological) state, microglial cells provide a certain extent of protection to the central nervous system. While in the activated (pathological) state, they may cause damage to central nervous system by secreting varieties of interleukins and cytokines that are closely related to inflammatory reactions and neuronal repair (17, 18). The pathological examination of PD demonstrated a large number of activated microglial cells in the SN, with an even higher intensity in the areas of neuronal degeneration (19, 20). In addition to neuronal damage in the brain of PD patients, the formation of Lewy body, accumulation of microglial cells, and repair of neurons enhanced the complexity of tissue structures and resulted in the increase of diffusion kurtosis parameters. Here, PD patients were not categorized into subtypes according to an early or late clinical period or in consideration of shorter or longer disease duration. The decrease observed in MK, Ka, and FA of deep GM may be attributed to neuronal damage. The increase of Kr in SN and GP may be due to the dominating effect of neuronal repair in the region.

DKI is an extension of DTI, which could reflect microstructural complexity, particularly in isotropic tissues such as gray matter (10). Some research focused on gray matter seemed more concerned with the overall diffusional metrics of MK, FA, and MD, rather than directional metrics such as axial diffusivity and radial kurtosis. This is because the GM microstructure lacks evident directionality that the radial and axial directions will be random and highly influenced by noise (21). However, a variety of changed Kr, Dr, Ka, and Kr in GM continue to be reported in normal aging (9–12, 22), in Moyamoya Disease (23), in Bipolar Disorders (24), and in various neurodegenerative processes including Alzheimer's disease (25, 26) and Parkinson's disease (27, 28). In fact, some of the deep GMs are composed of both gray matter and white matter (10, 29). The caudate nucleus includes the associative fibers projecting to the prefrontal cortex. The putamen contains projecting fibers from the primary motor and premotor cortices. Different from the caudate nucleus and putamen, the globus pallidus is rich in long, smooth, and sparsely branched neuronal dendrites that are well-protected by myelin. The red nucleus consists of densely packed cells and small myelinated axons (12, 30). These findings may suggest that varying proportion of white matter in the deep GM make motion of water restricted and directional. Actually, anisotropic water diffusion depends on oriented barriers. The deep GM is composed primarily of neurons and glia, which also have the highest iron deposits in the brain (12, 31). The myelinated axons, large cells, and ferritin protein constitute a complex microstructure that imposed extensive hindrance to free diffusion of water molecules. Thus, diffusion metrics in the deep GM appears to be different and more complicated than that in white matter. The degeneration of nigrostriatal dopaminergic neurons in the substantia nigra (SN) is the neuropathologic hallmark of PD (28). One previous study using the PD mouse model indicates that Dr in the SN is related with the numbers of SN dopaminergic neurons (32). As interesting findings in directional diffusion metrics were observed in deep GM, we speculate that they are of potential significance in deep GM. However, their underlying pathophysiological meaning should be examined in further studies. In our study, all diffusion parameters including the directional ones were presented to provide a comprehensive view with potentially intriguing findings.

### Diagnostic Efficiency of DKI Parameters

In our study, ROC analysis revealed that the MD for the globus pallidus had the best diagnostic performance. It is worth noting that, although the loss of dopaminergic neurons in the SN is the neuropathological hallmark in PD, the diffusion metric for the globus pallidus is the best biomarker to differentiate PD patients from HC. Globus pallidus is the most important relayed nucleus between striatum and subthalamic nuclei (STN) and plays an important role in the integration between inhibition from striatum and excitation from neocortex, thalamus, and STN (33). Changes in activity of neurotransmitters and related receptors along with abnormal synchronized oscillatory activity in the globus pallidus are associated with PD symptoms, such as bradykinesia, rigidity, and tremor, which reflects that globus pallidus plays an important role in the process of PD (34, 35). DBS researches have confirmed that stimulation of the globus pallidus is effective in reducing parkinsonian motor signs (36, 37). Among all the deep gray matter structures, globus pallidus has the relatively complex microstructural compositions and has the highest iron concentration in the brain (31). In summary, these results suggested that changed MD for the globus pallidus may reflect distinct pathological changes in PD. Future studies are needed to shed more light on the mechanisms underlying the changes in globus pallidus of patients with PD. Diffusivity parameters of RN and SN were useful; however, their sensitivity was not sufficiently high for clinical purposes. Alternatively, the diagnostic confidence was promoted with an accurate final diagnosis, considering that it is possible to employ these diagnostic parameters and that they may be applied in the form of assisted methods.

### MMSE Score

The correlation between DKI parameters and MMSE in our subjects suggested that the changes in the microstructure detected by DKI corresponded to the psycho-neural state; therefore, further investigation of DKI may benefit PD patients with dementia. As discussed previously, when the neuron repair dominated a particular region, the damage to the central neural system was restrained. This indicated that the psycho-neural state would be better in cases with a higher MMSE score. Diffusivity parameters represent the severity of damage that occurs to the brain, with higher levels indicating greater damage and a lower MMSE score. Therefore, both diffusivity and kurtosis parameters work as independent biomarkers to assess the psycho-neural state of PD patients. It was of great importance to note that the kurtosis parameters correlating with MMSE was focused on GP, RN, and SN, which had the most complicated microstructural compositions (12). We speculate that microstructural “complexity” in GP, RN, and SN may be the basis of psycho-neural state in PD. Meanwhile, negative correlations with MMSE score were observed for axial, radial, and mean diffusivity of the thalamus and HCN in our study. The caudate nucleus and thalamus had a relatively simple microstructure, which were mostly constituted by neurons and glia (12). The diffusion parameters of these regions likely reflect the distribution of neuron cell bodies, synapses, and dendrites. This may suggest that the integrity of thalamus and HCN had potential correlation with the psycho-neural state in PD.

## Conclusion

DKI can qualitatively distinguish PD from HC; furthermore, DKI-derived parameters can quantitatively evaluate the modifications of microstructures in extrapyramidal system gray matter nucleus in PD.

## Limitations

One limitation of our study is that the spatial resolution is 1.875 ×1.875 ×4 mm^3^. This may yield imperfect reference values and is thus biased. Another limitation is that treatment medication is not taken into account, the impact of which may lead to distorted brain structure. This needs to be explored in a future study.

## Data Availability Statement

The raw data supporting the conclusions of this article will be made available by the authors, without undue reservation, to any qualified researcher.

## Ethics Statement

The studies involving human participants were reviewed and approved by Ethics Committee of First Affiliated Hospital of Dalian Medical University. The patients/participants provided their written informed consent to participate in this study.

## Author Contributions

GB, MY, and LA conceived the study and participated in its design, data collection. TS, LY, and WW participated in statistical analysis, sketching ROI and drafting of the manuscript. SJ, SQ, and XL participated in image reconstruction and data analysis. ZY and DC participated in the manuscript revise, including statistical analysis. The authors read and approved the final manuscript.

## Conflict of Interest

The authors declare that the research was conducted in the absence of any commercial or financial relationships that could be construed as a potential conflict of interest.
