# Supplementary material for: Diffusion Kurtosis Imaging of Microstructural Changes in Gray Matter Nucleus in Parkinson Disease
Source: Front Neurol. 2020 Apr 17;11:252. doi: 10.3389/fneur.2020.00252 (PMC7180218; doi:10.3389/fneur.2020.00252)

**Supplementary Table S1: The ICC values of all ROIs between the two neurologists.**

|  | Globus pallidus | | Substantia nigra | | Red nucleus | | Putamen | | Head of caudate nucleus | | Thalamus | |
| --- | --- | --- | --- | --- | --- | --- | --- | --- | --- | --- | --- | --- |
|  | ICC | 95% CI | ICC | 95% CI | ICC | 95% CI | ICC | 95% CI | ICC | 95% CI | ICC | 95% CI |
| MD | 0.79 | 0.54-0.91 | 0.76 | 0.48-0.90 | 0.84 | 0.64-0.94 | 0.78 | 0.51-0.91 | 0.88 | 0.71-0.95 | 0.75 | 0.47-0.89 |
| Da | 0.88 | 0.71-0.95 | 0.78 | 0.52-0.91 | 0.92 | 0.81-0.97 | 0.88 | 0.72-0.95 | 0.83 | 0.62-0.93 | 0.79 | 0.53-0.91 |
| Dr | 0.91 | 0.77-0.96 | 0.79 | 0.54-0.92 | 0.85 | 0.66-0.94 | 0.77 | 0.49-0.90 | 0.80 | 0.55-0.92 | 0.80 | 0.55-0.92 |
| FA | 0.92 | 0.80-0.97 | 0.86 | 0.67-0.94 | 0.90 | 0.75-0.96 | 0.90 | 0.77-0.96 | 0.95 | 0.87-0.98 | 0.91 | 0.77-0.96 |
| MK | 0.91 | 0.79-0.97 | 0.87 | 0.70-0.95 | 0.91 | 0.78-0.96 | 0.93 | 0.81-0.97 | 0.80 | 0.56-0.92 | 0.89 | 0.76-0.96 |
| Ka | 0.92 | 0.80-0.97 | 0.93 | 0.83-0.97 | 0.86 | 0.71-0.95 | 0.88 | 0.71-0.95 | 0.86 | 0.68-0.94 | 0.79 | 0.50-0.92 |
| Kr | 0.93 | 0.79-0.97 | 0.91 | 0.77-0.96 | 0.91 | 0.78-0.96 | 0.79 | 0.55-0.92 | 0.86 | 0.67-0.94 | 0.93 | 0.83-0.97 |

**Supplementary Table S2: the meanings of the diffusion kurtosis/tensor parameters**

| Diffusion kurtosis/tensor  parameters | Definition | Increasing | Decreasing |
| --- | --- | --- | --- |
| MK | The average of the kurtosis over all possible diffusion directions(M, I et al. 2012). Equal to 0 to 3. | High complexity of tissue structure; non-gaussian distribution of water molecules diffusion; restricted diffusion; such as myelination, diameter of axon, cytotoxicity edema. | Low complexity of tissue structure; water molecules diffusion tends to gaussian distribution; unrestricted diffusion; such as low-grade astrocytoma compared to glioblastoma. |
| Ka | The kurtosis in the direction of the diffusion tensor eigenvector with the largest diffusion eigenvalue. | Water molecules in the direction parallel to the white matter have obviously restricted diffusion. | Water molecules in the direction parallel to the white matter have mildly restricted diffusion. |
| Kr | The average of the kurtosis over all directions perpendicular to the diffusion eigenvector with the largest eigenvalues. | The non-gaussian distribution water molecules in the direction perpendicular to the white matter have obviously restricted diffusion. White matter is higher than gray matter. | The non-gaussian distribution water molecules in the direction perpendicular to the white matter have mildly restricted diffusion. |
| MD | The overall  mean-squared displacement of molecules (average ellipsoid size) and the overall presence of obstacles to diffusion. | Water molecules have strong diffusivity. Relevant to intercellular space, extracellular edema, apoptosis and necrosis, demyelination, etc. | Water molecules have week diffusivity. Relevant to cell density, cell atypia, intracellular edema, gliosis, etc. |
| Da | Diffusion ability in the direction parallel to the long axis of the axon. | Water molecules have strong diffusivity. | Water molecules have week diffusivity. |
| Dr | Diffusion ability in the direction perpendicular to the long axis of the axon. | Water molecules have strong diffusivity. | Water molecules have week diffusivity. |

**Supplementary Figure S1: the placement of ROI**


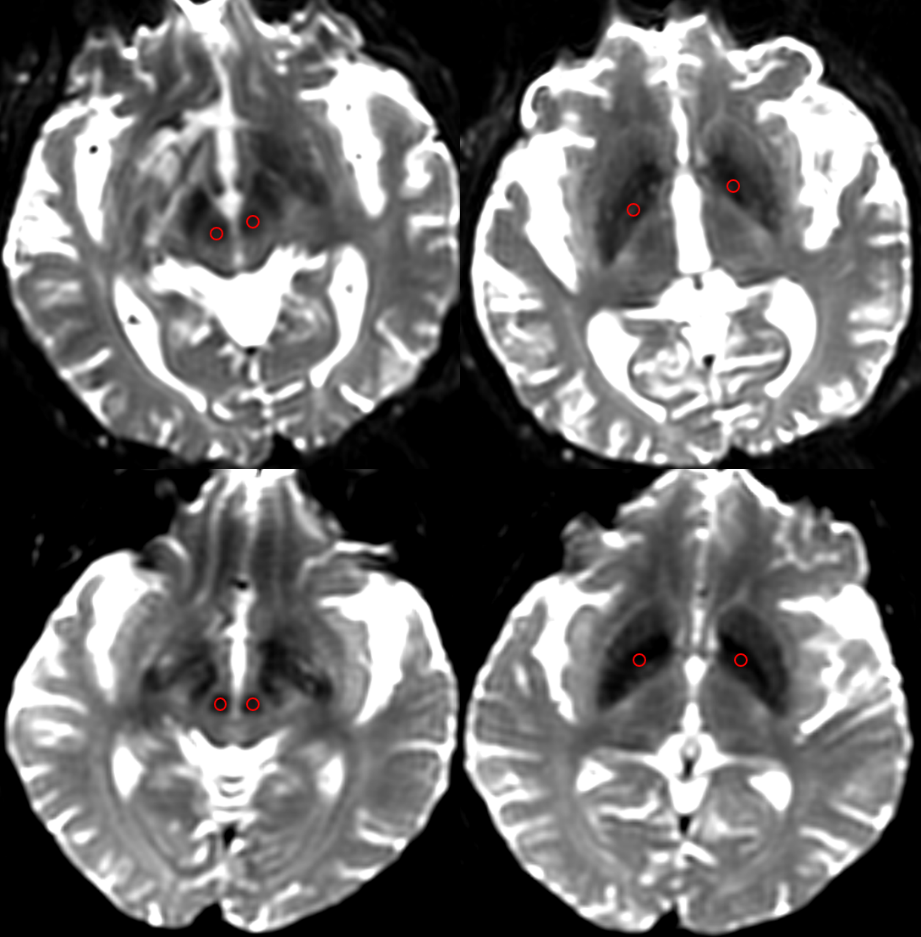

Supplement: Supplementary file 1 [file Data_Sheet_1.docx]
